# Immunomodulatory Effects of Mesenchymal Stem Cells on Drug-Induced Acute Kidney Injury

**DOI:** 10.3389/fimmu.2021.683003

**Published:** 2021-06-04

**Authors:** Qiuxia Han, Xiaochen Wang, Xiaonan Ding, Jun He, Guangyan Cai, Hanyu Zhu

**Affiliations:** ^1^ Department of Nephrology, First Medical Center of Chinese People’s Liberation Army (PLA) General Hospital, Nephrology Institute of the Chinese People’s Liberation Army, State Key Laboratory of Kidney Diseases, National Clinical Research Center for Kidney Diseases, Beijing Key Laboratory of Kidney Disease Research, Beijing, China; ^2^ School of Medicine, Nankai University, Tianjin, China; ^3^ Department of Genetics, Changsha Hospital for Maternal and Child Health Care, Hunan, China

**Keywords:** acute kidney injury, mesenchymal stem cell, immunomodulation, cell therapy, kidney repair, drug-induced AKI, ischemia-reperfusion

## Abstract

Drug-induced nephrotoxicity is an important and increasing cause of acute kidney injury (AKI), which accounts for approximately 20% of hospitalized patients. Previous reviews studies on immunity and AKI focused mainly on ischemia-reperfusion (IR), whereas no systematic review addressing drug-induced AKI and its related immune mechanisms is available. Recent studies have provided a deeper understanding on the mechanisms of drug-induced AKI, among which acute tubular interstitial injury induced by the breakdown of innate immunity was reported to play an important role. Emerging research on mesenchymal stem cell (MSC) therapy has revealed its potential as treatment for drug-induced AKI. MSCs can inhibit kidney damage by regulating the innate immune balance, promoting kidney repair, and preventing kidney fibrosis. However, it is important to note that there are various sources of MSCs, which impacts on the immunomodulatory ability of the cells. This review aims to address the immune pathogenesis of drug-induced AKI versus that of IR-induced AKI, and to explore the immunomodulatory effects and therapeutic potential of MSCs for drug-induced AKI.

## Introduction

In the past decade, pharmaceutical companies have developed many life-saving drugs for patients with cancer. Unfortunately, some of these drugs are associated with nephrotoxicity, which remains an important and more frequent cause of acute kidney injury (AKI). Currently, AKI represents a serious global public health challenge, with significant adverse economic and medical burden, that affects approximately 13.3 million people annually (1, 2). Drug-induced AKI is a particularly important category, accounting for 19–26% of AKI cases (2). The use of nephrotoxic drugs is the main cause of severe AKI in critically ill patients. Moreover, drug-induced nephrotoxicity is one of the main factors leading to unsuccessful drug development, leading to loss of money and time in the pharmaceutical industry (3). Notably, approximately 19% of phase 3 clinical trial failures are caused by nephrotoxicity (3).

Recent studies have provided a deeper understanding of the mechanisms underlying drug-induced AKI. In particular, oxidative stress was shown to activate inflammatory responses, by promoting the release of pro-inflammatory cytokines and the accumulation of inflammatory cells in tissues, which supports the progression of AKI (4). Mesenchymal stem cell (MSC) therapy has been considered to hold great potential for the treatment of drug-induced AKI given its powerful immune regulatory effects (5). Previous studies on the immune pathogenesis of AKI and MSC therapy mostly focused on ischemia-reperfusion (IR), but drug-induced AKI is also important and needs more attention. The immune pathogenesis of IR and drug-induced AKI were reported to be different, which may also impact on the efficiency of MSC treatment on drug-induced AKI. Therefore, the differences between these two types of AKI could provide a good reference for scientists to explore MSC-based treatment strategies for drug-induced AKI in the future. In addition, it is very important to notice that MSCs can be derived from various sources and their immunomodulatory ability may differ based on their origin. When using MSCs for clinical treatment of patients, it should be considered the difficulty of obtaining these cells, as well as the number of cells and ethical limitations. Therefore, understanding the immunomodulatory ability, proliferative potential, and clinical application characteristics of MSCs from different sources can aid to choose the most appropriate MSC population for clinical use in AKI treatment.

In this review, we provide an in-depth, comprehensive summary of the immune pathogenesis of drug-induced AKI and subsequent molecular mechanisms, including the different types of immune cells, cytokines, and related signaling pathways. The immunomodulatory effects of MSCs for the treatment of drug-induced AKI were also explored based on the latest research progress. In particular, we compared the different immune pathogenesis of drug-induced and IR-induced AKI, as well as the different immune regulatory abilities of MSCs from different sources.

## Drug-induced AKI and Common Nephrotoxic Drugs: The Cisplatin Example

Several recent, large-scale epidemiological studies have shown that nephrotoxic drugs cause severe acute renal failure in many critically ill patients (2), for whom the use of potentially nephrotoxic drugs is, in most cases, unavoidable (6). Drug-induced AKI usually presents as one of two types: acute tubular necrosis, which is directly related to nephrotoxicity to the renal tubular epithelial cells, or immune-mediated acute interstitial nephritis, which develops from drugs that cause allergic reactions. Approximately 60% of patients with hospital-acquired AKI experience nephrotoxic induced by medications (7). Among the various cellular mechanisms of drug-induced AKI, the most important is the accumulation of inflammatory cells in the tissue, which in turn activates the inflammatory response and oxidative stress that triggers the release of pro-inflammatory factors. Nephrotoxic injuries often occur in the proximal renal tubules due to the administration of cisplatin, aminoglycosides (gentamycin, kanamycin, streptomycin, or tobramycin), amphotericin B, antiviral agents (adefovir, cidofovir, or tenofovir), radiocontrast, bisphosphonate, among other drugs (4).

Cisplatin is a chemotherapy drug, and is the most classic and commonly used model for basic research on drug-induced AKI, with a reported frequency of cisplatin-induced AKI ranging from 20% to 30% due to dose-limiting side effects in the treatment of various solid cancers, including testicular, ovarian, cervical, breast, lung, head and neck, bladder, and gastric cancer (8). Although there are other less common cisplatin-induced toxicities (such as ototoxicity, gastrotoxicity, and myelosuppression), nephrotoxicity has a major impact on the patient health and should be the first side effect to be monitored and evaluated in patients undergoing cancer chemotherapy. Notably, the accumulation of toxic intermediates in the kidney limits the use of cisplatin, which leads to incomplete chemotherapy. Therefore, the mechanism reviewed hereafter is mainly based on basic research conducted with the cisplatin-induced AKI model.

The typical histological features of drug-induced AKI are severe tubule damage comprising lumen dilation, significant cytoplasmic simplification, increased cytoplasmic eosinophilia, and disappearance of brush borders and of the tubule epithelium. First, several events, such as loss of polarity and cytoskeletal integrity, and cell necrosis and apoptosis, occur in the proximal tubular epithelial cells of the kidney over time (9, 10). Subsequently, necrosis induces inflammation, and damage-associated molecular patterns (DAMPs) and other pro-inflammatory molecules are released, resulting in the activation of the innate immune system *via* membrane surface receptors. Inflammatory cells, such as white blood cells, are recruited into the peritubular interstitium. Furthermore, inflammation accelerates the damage to the renal tubular tissues and causes necroptosis and the release of tumor necrosis factor alpha (TNF-α) and other inflammatory factors that continue to drive cell necrosis. This leads to tubular necrosis and renal insufficiency, forming an inflammation-necrosis amplification loop (11, 12). Another mechanism of drug-induced AKI is oxidative stress. Drug nephrotoxicity directly acts on the proximal renal tubules and causes cell damage, such as mitochondrial dysfunction, lysosomal hydrolase inhibition, phospholipid damage, and increased intracellular calcium concentrations, thereby leading to the formation of reactive oxygen species (ROS) (13). The pathogenic mechanisms of ROS have three main aspects: first, nephrotoxic drugs react with cellular antioxidants (such as glutathione) when they are in a highly reactive form (14, 15), thus, depleting or inactivating them, leading to the accumulation of endogenous ROS in cells. ROS activates the intracellular mitogen-activated protein kinases, p53, p21, and other pathways, leading to the death of renal tubular cells. Second, ROS directly or indirectly promotes fibrosis by promoting tissue inflammation. Fibrosis and inflammation will, in turn, promote positive feedback pathways, further increasing ROS production and stimulating the secretion of inflammatory factors. Third, nephrotoxic drugs affect the normal respiration of mitochondria, making them dysfunctional and increasing the production of ROS (16).

## Different Immune Mechanisms of Drug-Induced and IR-Induced AKI

AKI is mainly triggered by IR injury, which causes high morbidity and mortality in both adults and children (17). IR-induced AKI results from acute hypoxia caused by reduced blood perfusion in the renal tissue, which is prone to occur in the renal tubule region. Reperfusion leads to the production of metabolites, such as nitric oxide and ROS, which can damage the cell membranes and lead to cell apoptosis. However, drug-induced AKI is more common in infants and older people with underlying cardiovascular diseases and renal dysfunction, such as intravascular volume depletion, diabetes, congestive heart failure, chronic kidney disease, and sepsis (18, 19). Drug-induced renal injury, which results from the direct damage to the renal tubular epithelial cells, occurs when the increasing concentration of nephrotoxic drugs in the renal tubule reaches a toxic level. Therefore, the degree of damage is related to the drug dose administrated. Noteworthy, there are several differences in the pathogenesis of IR-induced and drug-induced AKI; however, there are very limited systematic reviews comparing the differences in the pathogenesis between these two models. Understanding the differences in their immune pathogenesis may be helpful for the management of AKI. A summary of these differences is provided in **Table 1**.

**Table 1:** Comparison of the immune mechanisms between drug-induced and ischemia reperfusion-induced AKI.

Immune system component	Mechanism of IR-induced AKI (e.g., organ grafting)	Mechanism of drug-induced AKI (e.g., cisplatin)
T cells	CD8^+^ cells have no obvious pathogenic effect in IR injury (20).	Inhibition of CD8^+^ can significantly improve kidney damage (20).
Dendritic cells	Higher proportion of mature dendritic cells, antigen presentation effect and pro-inflammatory response (21, 22).	Higher proportion of immature dendritic cells, promote kidney repair (23).
Neutrophils	Infiltration at the injury site, but inhibition of neutrophils can significantly reduce kidney damage (24).	Infiltration at the injury site, but inhibition of neutrophils cannot significantly reduce kidney damage (25).
Macrophages	Macrophages have similar actions in drug-induced and IR-induced AKI. M1 cells are the dominant cells in damage stage; M2 cells play a role in tissue repair (26).	
Complement system	The mechanism of action of the complement system in drug-induced and IR-induced AKI involves the activation of C5a/C5aR–NF-κB pathway (27).	
Cytokines/pathway	The levels of IL-11 increase (28). Inhibition of IL-18 can significantly prevent kidney damage (29). CCL5 and IL-1α slightly increase (28). Inhibition of TNF-α cannot significantly reduce kidney damage (28). NLRP3 pathway is the key pathogenesis of inflammation (30).	The levels of IL-11 do not increase (28). Inhibition of IL-18 cannot significantly prevent kidney damage (29). CCL5 and IL-1α significantly increase (28). Inhibition of TNF-α can significantly reduce kidney damage (28). NLRP3 pathway is less important (30).

AKI, acute kidney injury; C5a, complement component 5a; C5aR, complement component 5a receptor; CCL5, C-C motif chemokine ligand 5; IL, interleukin; IR, ischemia-reperfusion; M1, pro-inflammatory macrophages; M2, anti-inflammatory macrophages; NF-κB, nuclear factor kappa B; NLRP3, NLR family pyrin domain containing 3; TNF, tumor necrosis factor.

T cells play an important role in AKI. In IR-induced AKI, CD8^+^ cells have no obvious pathogenic effects. However, in the cisplatin-induced AKI model, CD8-deficient mice had significantly less damage than wild-type mice, indicating that CD8^+^ cells have an important pathogenic role in this model (20). No obvious differences in other subpopulations of T cells were found between the two models. During the inflammatory response to IR injury, the main role of renal dendritic cells (DCs) is to serve as antigen-presenting cells to T cells, release TNF-α, upregulate adhesion molecules, and promote leukocyte extravasation (21, 22). However, renal DCs play a protective role in drug-induced AKI. In one study, infiltration of DCs in damaged tissues resulted in secretion of cytokines such as interleukin (IL)-10 to prevent AKI progression (23). In the IR model, inhibition of neutrophil recruitment can significantly reduce kidney damage, which suggests that neutrophil infiltration is one of the pathogenic mechanisms of renal IR injury (24). In contrast, inhibition of neutrophil infiltration has no effect on cisplatin-induced kidney injury, indicating that neutrophil infiltration is not necessary for cisplatin-induced kidney damage (25). Importantly, macrophages have pro-inflammatory (M1) and anti-inflammatory (M2) cell phenotypes and their roles in the two AKI models are similar (26), with M1 cells being dominant in the damage stage, whereas M2 cells playing an important role in the repair stage. In addition, the relationship between the complement system and AKI has been studied more in the IR injury model than in the drug-induced AKI model. However, the mechanism of action of the complement system in both models is mainly mediated by the activation of the complement component 5a and its receptor (C5a/C5aR)–nuclear factor kappa B (NF-κB) pathway (27).

Cytokines and chemokines are differently expressed in drug-induced and IR-induced AKI, which may be due to the different cellular origins and actions of the molecules. IL-11 is upregulated in IR-induced (but not in cisplatin-induced) AKI (28). In addition, inhibition of IL-18 can significantly reduce IR-induced renal injury but not cisplatin-induced nephrotoxicity (29), which suggests that IL-18 may be an important factor in IR-induced AKI. Moreover, the expression of the C-C motif chemokine ligand (CCL) 5 and IL-1α is significantly increased in cisplatin-induced AKI, but only slightly increased in the IR injury model (28). Furthermore, TNF-α-deficient mice are resistant to cisplatin nephrotoxicity, thereby suggesting that TNF-α plays an important role in the pathogenesis of cisplatin-induced renal injury. Accordingly, TNF-α inhibitors can ameliorate renal dysfunction and reduce the structural damage caused by cisplatin, and inhibition of TNF-α more effectively reduce cisplatin-induced than IR-induced kidney damage (28). In addition, the nucleotide-binding oligomerization domain-like receptors (NLR) family pyrin domain containing 3 (NLRP3) pathway is critical in the pathogenesis of IR-induced AKI, but is less important in cisplatin-induced AKI (30).

## Different immunoregulation capability of MSCs from various origins

MSCs are pluripotent stem cells with anti-inflammatory and immune tolerance potential (31), which were first identified in the bone marrow stroma where supported the development and differentiation of hematopoietic stem cells. In recent years, with research advances, MSCs have been increasingly used in clinical practice given their immunosuppressive functions and tissue repair ability (32). Hence, stem cell therapy is another medical revolution that may be considered after drug therapy and surgical treatment. MSCs can easily expand *in vitro* into high numbers in a short period of time. This is an important prerequisite for MSCs that are widely used in experimental research and clinical practice, including AKI treatment (33). Moreover, MSCs can be cultured from adipose tissue, cord blood, umbilical cord, placenta, and fetal lungs. However, the biological characteristics of the MSCs originating from these various tissues are different, especially concerning their immune regulation capacity (34). The immunological activity of MSCs from different tissues may differ because of the different original activation states of these cells in the source tissues (35–37). The differences in the immunomodulatory ability, proliferation potential, and clinical application characteristics of MSCs from different sources is summarized in **Table 2**.

**Table 2:** Immunomodulatory ability, proliferation potential, and clinical application characteristics of MSCs from different sources.

Source	Immunomodulatory ability^1^	Proliferation potential^1^	Clinical application characteristics
Bone marrow	+	+	The related research started earlier and is more thorough. The number of cells that can be extracted is relatively small. The passage ability that can maintain the characteristics is weak.
Umbilical cord	+++	+++	The number of extractable cells is obviously more than that of bone marrow. Strong ability of passage. No ethical limit.
Placenta	+++	+++	The number of extractable cells is more than that of umbilical cord and bone marrow. No ethical limit.
Adipose tissue	++	++	Easy to get relatively large number of cells from rich resource of adipose tissue. Whether the cells can adapt to the environment *in vivo* remains to be further studied.

^1^Higher number of + represents a stronger degree.

Current studies have shown that the immunological activity of MSCs originating from different tissues is “strong” or “weak” rather than “present” or “absent” (38). Yoo et al. compared the immune regulatory functions of adipose-derived MSCs (AD-MSCs), umbilical cord blood-derived MSCs, umbilical cord-derived MSCs (UC-MSCs), and bone marrow-derived MSCs (BM-MSCs) on T lymphocytes (34), and found that all four types of MSCs inhibited the proliferation of activated T cells and the secretion of interferon-γ and TNF-α. Moreover, Bochev et al. found that both BM-MSCs and AD-MSCs could inhibit the secretion of immunoglobulins by activating B lymphocytes (39). However, the inhibitory effects of AD-MSCs on immunoglobulin secretion were stronger than those of BM-MSCs. Some study also reported that AD-MSCs have higher indoleamine 2,3-dioxygenase (IDO) activity and secret more IL-6, IL-10, and transforming growth factor (TGF)-β than BM-MSCs (35, 40). Moreover, Barcia et al. found that compared with BM-MSCs, UC-MSCs have stronger immunomodulatory abilities, can more efficiently inhibit CD3- and CD28-induced lymphocyte proliferation, and can more efficiently induce the production of CD3^+^CD4^+^CD25^+^Foxp3^+^ regulatory T cells (Tregs) (41).

Nevertheless, some studies have shown that the *in vitro* expansion capabilities of MSCs from different tissues are not completely the same. Different microenvironments may be the reason for these differences. Li et al. compared the proliferative capacity of BM-MSCs and AD-MSCs, and found that AD-MSCs have stronger proliferation potential (42). However, as the age of the donor increases, the number and proliferation capacity of BM-MSCs and AD-MSCs significantly decreases. MSCs derived from perinatal tissues (such as umbilical cord, placental chorion, and amniotic membrane) have much stronger proliferation potential than BM-MSCs. Comparing MSCs isolated from umbilical cord tissue and bone marrow, more MSCs can be obtained from umbilical cord tissues, and with greater proliferation ability than BM-MSCs (43). After 30 generations, the proliferative capacity of UC-MSCs did not change significantly, whereas BM-MS showed weakened proliferation ability and prolonged doubling time after six passages.

In summary, perinatal MSCs (MSCs derived from the umbilical cord and placenta) generally have higher immunomodulatory capabilities, while BM-MSCs show higher potential to support the regeneration process, such as neuronal differentiation and development (43). These differences between perinatal and bone marrow-derived MSCs may impact on their clinical application. In addition, when MSCs are used in the clinical treatment of diseases, the feasibility of its acquisition, the number of cells, and ethical limitations must be considered. For example, BM-MSCs and AD-MSCs have several limitations. They are generally obtained from adults and the number of cells available is limited. Moreover, there are restrictions regarding the age of the donor. However, MSCs in the perinatal period have stronger expansion capacity and have no ethical restrictions. After expansion, the basic properties and morphology of MSCs have no obvious changes, so that a large number of MSCs can be obtained from the same sample to meet the needs of the clinical cell therapy (43). Therefore, compared with adult-derived stem cells that proliferate slowly, perinatal MSCs have unique advantages. From this perspective, MSCs derived from umbilical cord and placenta are an ideal choice.

## Immunomodulatory Effects of MSCs on Drug-Induced AKI

The strong ability of MSCs to differentiate is one of the main mechanisms contributing for tissue damage repair. Although the initial focus on MSCs was on their regenerative ability to differentiate into various tissue cell types (33), more attention is currently addressed onto their ability to regulate immune responses (44). *In vivo* and *in vitro* studies have shown that MSCs can directly inhibit the proliferation, differentiation, and effector mechanisms of T cells by preventing the externalization and maturation of DCs. In addition to T cells and DCs, the target cells of MSCs include many other types of immune cells, including natural killer cells, B cells, neutrophils, and macrophages (45). MSCs regulate the function of these cells, including the secretion of cytokines and their subsequent cytotoxic effects, eventually exerting anti-inflammatory effects and/or inducing an immune tolerance state. To date, the immune regulation mechanisms of MSCs remain poorly understood, but it is generally believed that MSCs have an immunosuppressive function mainly through cell-to-cell contact with a variety of immune cells and secretion of soluble factors, such as prostaglandin E2 (PGE2) and IDO, thereby reducing tissue damage caused by inflammation (46). MSCs have low immunogenicity, that is, MSCs do not express major histocompatibility complex class II molecules or costimulatory molecules, and do not cause the activation and proliferation of allogeneic lymphocytes, which provides the possibility for the clinical application of allogeneic MSCs transplantation (47). These cells inhibit the proliferation of activated T lymphocytes and the secretion of interferon-γ, the proliferation of B lymphocytes and the secretion of immunoglobulins, the killing activity of natural killer cells, the differentiation of M1 macrophages, the secretion of pro-inflammatory factors, and the differentiation of monocytes into DCs (48).

**Figure 1:**
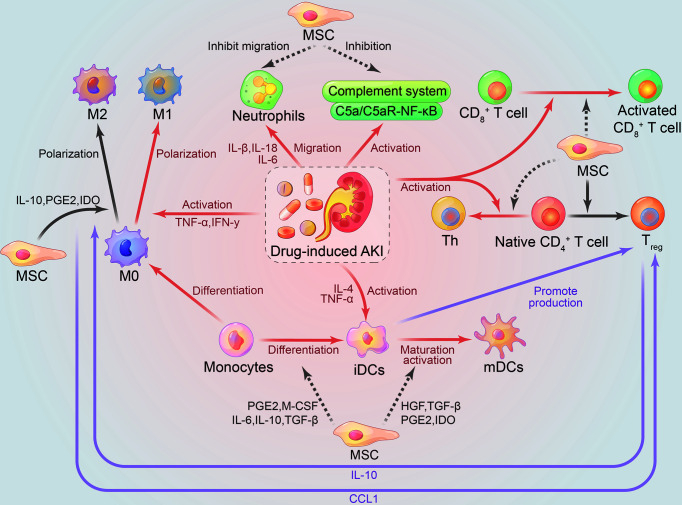
Immunomodulatory effects of mesenchymal stem cells on drug-induced AKI. Gray, therapeutic effect of mesenchymal stem cells on the kidney; Red, damage effect of AKI on the kidney; Purple, interaction between immune cells; Solid line, promoting effect; dashed line, inhibiting effect. AKI, acute kidney injury; iDCs, immature dendritic cells; mDCs, mature dendritic cells; Th, helper T cells; Treg, CD3^+^CD4^+^CD25^+^Foxp3^+^ regulatory T cells; HGF, hepatocyte growth factor; IDO, indoleamine 2,3-dioxygenase; PGE2, prostaglandin E2; TGF-β, transforming growth factor β.

There is a very close connection between the immune system and the kidney system (49). Pro-inflammatory DAMPs, pathogen-associated molecular patterns (PAMPs), toll-like receptors (TLRs), oxidative stress, complement system, resident DCs, neutrophils, T lymphocytes, macrophages, and secreted cytokines and chemokines, all participate in the immunopathological mechanism of drug-induced AKI and promote disease progression (20, 28, 50–53). Therefore, imbalance of the immune system has a direct or indirect impact on the progression of kidney disease. If the immunopathological process of AKI continues, this may lead to renal fibrosis and/or chronic kidney disease. The immunomodulatory mechanism of MSCs in AKI treatment is shown in **Figure 1**.

## The Immune Microenvironment of Drug-Induced AKI

### T Cells

Approximately 30% of the circulating white blood cells in healthy adults are T cells from the thymus (51). T cells are important immune regulators of drug-induced AKI, as T cell infiltration was observed in damaged renal tissues within 1 h after cisplatin administration, which reached a peak at 12 h and decreased after 24 h (54). Compared with wild-type mice, T cell knockout significantly improved renal function and prolonged survival in cisplatin-induced AKI mice. Furthermore, infiltration of neutrophils and macrophages into the kidney was reduced in T cell knockout mice, suggesting that T cells play a critical role in the recruitment of immune cells to the site of injury. CD4^+^ T cells are activated and quickly infiltrate the damaged kidneys, mediating further renal tissue damage (54, 55). Depending on the regulation of the immune microenvironment, naive CD4^+^ cells can differentiate into helper T cells (Th) or Tregs, and play different roles. Cisplatin nephrotoxicity causes naïve CD4^+^ T cells to differentiate into Th, resulting in a strong immune response and kidney damage. Tregs are believed to play a protective role against cisplatin-induced AKI. They enter the kidney tissue and directly modulate the response of cells to inhibit the pro-inflammatory signals promoted by cisplatin toxicity, reduce renal macrophage infiltration, as well as the levels of TNF-α and IL-1β, thereby reducing renal dysfunction. CD4^+^ T cells complete the immune response to cisplatin through the paracrine pathway (secreting TNF-α, IL-17, IL-33, and IL-10), whereas cytotoxic CD8^+^ T cells induce renal cell injury in a contact-dependent manner (54). Cisplatin can upregulate the expression of Fas receptors on damaged renal tubular epithelial cells, allowing it to interact with Fas ligands on CD8^+^ T cells infiltrating the kidney, and then trigger the apoptosis of damaged cells (49). MSCs can inhibit the production and activation of Th and CD8^+^ T cells, and reduce inflammation in cisplatin-induced AKI. Furthermore, MSCs promote the differentiation of naïve CD4^+^ T cells into Tregs by secreting hepatocyte growth factor, TGF-β, PGE2, and IDO, which in turn secrete IL-10. Moreover, PGE2 and IDO secreted by MSCs can promote the differentiation of macrophages into the anti-inflammatory M2 phenotype (56). Therefore, the anti-inflammatory effects of MSCs can be further improved, and the kidney damage caused by cisplatin can be reduced.

### Dendritic Cells

DCs are the most common immune cells that maintain renal homeostasis. Renal DCs are located between the renal tubules and the peritubular capillaries. They act as effector cells and intermediates between endothelial and epithelial cells (21). In the injured state, renal DCs express specific cytokines through toll-like receptors, nod-like receptors, cell fragments, or other DAMPs, and present antigens to T cells to complete the transition from innate to adaptive immunity (57, 58). Similar to macrophages, DCs have two types: a pro-inflammatory phenotype (mature DCs; mDCs) and an anti-inflammatory phenotype (immature DCs; iDCs). Renal DCs are capable of immune induction and/or immune tolerance; thus, imbalanced immune regulation leads to the occurrence and progression of AKI. In cisplatin-induced AKI, IL-4 and TNF-α activate iDCs into mature phenotypes at the initial stage, causing kidney damage. IL-10, which is mainly derived from renal iDCs and plays an important role in immune regulation, has a nephroprotective effect and can reduce the nephrotoxicity of cisplatin in an inducible nitric oxide synthase-dependent manner (59). Hence, inhibition of nitric oxide synthase activity leads to a reduced number of IL-10-secreting DCs and loss of the renal protective effect of BM-MSCs (60). Furthermore, clearance of IL-10-secreting DCs can aggravate cisplatin-induced nephrotoxicity (52, 59). MSCs can inhibit the maturation of DCs and promote their transition to a tolerant immunosuppressive phenotype. They can also inhibit the differentiation of monocytes into iDCs by secreting PGE2, monocyte colony-stimulating factor, IL-6, IL-10, and TGF-β. Furthermore, MSCs can inhibit the maturation of iDCs into mDCs by secreting hepatocyte growth factor, TGF-β, PGE2, and IDO (56). PGE2 can inhibit the production and secretion of osteopontin, a cytokine released from DCs, which contributes to tissue inflammation (61). In the inflammatory microenvironment, iDCs stimulated by IL-10 (produced by MSCs) fail to express markers of mDCs (62). These iDCs do not produce TNF-α and, thus, can inhibit the progression of inflammation at the site of injury (63, 64). In addition, iDCs can promote the production of Tregs and exert anti-inflammatory effects. Some studies have shown that MSCs reduce the expression of major histocompatibility complex class II, CD40, and CD86 costimulatory molecules by mDCs, which is the cause of the decrease in T cell proliferation and weaker immune responses (65). MSCs also inhibit the antigen-presenting effects of DCs on CD4^+^ and CD8^+^ T cells in inflammatory lymph nodes (66), and reduce the production of pro-inflammatory factors, such as interferon-γ and IL-17 (67). These immunomodulatory effects help to reduce cisplatin-induced kidney damage.

### Neutrophilic Granulocytes

When AKI occurs, many neutrophils are transported and accumulate in the blood vessels of the kidney, leading to blockage of the microvascular network. With the help of endothelial chemokines and adhesion molecules, neutrophils are attracted to the damaged site, mediate inflammation, and assist in the elimination of damaged cells in preparation for the repair of the kidney (68). However, this effect is a double-edged sword. If the inflammatory response is insufficient, damaged or dead cells cannot be cleared in a timely manner, resulting in delayed tissue healing and AKI may eventually develop into chronic kidney disease. However, if the inflammatory response is excessive, cytotoxic compounds (ROS, proteases, and inflammatory cytokines) secreted by neutrophils will adhere to the kidney endothelial cells in large quantities, which triggers inflammatory factor infiltration and impairs the cell repair processes. A study reported that neutrophil infiltration increased in a group of cisplatin-treated mice compared to the control group (25). Interestingly, when caspase-11 was inhibited, neutrophil infiltration decreased, suggesting that cisplatin can induce neutrophil infiltration *via* caspase-11 activation (69). Other ways to significantly reduce neutrophil infiltration after administration of cisplatin include the use of TNF-α inhibitors, TLR-4 antagonists, or anti-intercellular adhesion molecule-1 antibodies (53), thereby partially improving cisplatin-induced AKI. Studies have shown that when MSCs are used to treat AKI, the migration and infiltration of neutrophils in the kidneys, as well as the serum creatinine and blood urea nitrogen levels, are significantly reduced, and renal function and kidney pathological damage is ameliorated (31, 70).

### Macrophages

The mononuclear-macrophage system plays a crucial role in the immune response. According to changes in the microenvironment at the injury site, macrophages can differentiate into pro-inflammatory (M1) or anti-inflammatory (M2) cell phenotypes, which play an important role in the stage of damage and repair, respectively (26). After acute renal tissue destruction, monocytes are immediately recruited to the damaged site and are stimulated by DAMPs and PAMPs (TNF-α, interferon-γ, saturated fatty acids, and IL-6) to differentiate into the M1 phenotype and participate in the innate immune response (71, 72). Additionally, through the production and secretion of various cytotoxic substances, such as chemokines, pro-inflammatory cytokines, and inducible nitric oxide synthase, M1 cells amplify the renal inflammatory response in AKI and exacerbate disease progression (50). However, blockage of macrophage recruitment does not completely prevent cisplatin-induced AKI (73). Some studies have shown that the M2 phenotype inhibits excessive inflammatory reactions by secreting anti-inflammatory mediators (such as IL-10 and TGF-β), and promotes renal cell repair (74, 75). MSCs therapy can significantly reduce cisplatin-mediated immune cell infiltration in damaged kidney tissues, such as macrophages, DCs, neutrophils, and CD4^+^ and CD8^+^ T cells. As a result, it inhibits the production of inflammatory factors, but stimulates the secretion of the anti-inflammatory factors IL-10, IL-6, nitric oxide, and kynurenine (60). In addition, MSCs can promote the differentiation of macrophages into the anti-inflammatory M2 phenotype by secreting various factors including PGE2, IL-10, and IDO (67). Furthermore, the M2 phenotype can secrete CCL1 to promote Treg production. Therefore, the anti-inflammatory effects of MSCs can be further improved, and the kidney damage caused by cisplatin can be ameliorated.

### Complement System

The complement system plays an important role in innate immunity and is activated by either of three pathways: classical, alternative, or lectin pathways (76), which eventually lead to the cleavage of C5 into C5a and C5b. These two allergenic proteins later form the complement membrane in the first step of forming the attack complex. Studies have shown that N-acetylcysteine can reduce the nephrotoxic effect of cisplatin by inhibiting the binding of C5a to C5aR. In addition, inhibiting C5aR can reduce neutrophil infiltration and the effects of inflammatory factors (77). When MSCs were used to treat AKI, serum C5a levels and C5aR expression in renal tissues were significantly reduced, and NF-κB translocation was also reduced. Hence, MSCs can reduce AKI by inhibiting the activation of the C5a/C5aR–NF-κB pathway (27).

## Conclusion and Perspective

To date, the safest route of administration of MSCs in clinical applications is through vascular injection; thus, the number of MSCs that can reach the kidney is very small (47). Several scientists have attempted to improve the efficacy of MSCs through various methods. Improving the immunomodulatory ability of MSCs is key to improving their therapeutic effect. Studies have been conducted to regulate the relevant immune responses by innovative pretreatment methods, thereby enhancing the kidney repairing effect (78, 79). Currently, most reviews focus on AKI due to ischemia-reperfusion. However, there are relatively few studies on drug-induced AKI, especially on the role of the complement system. Drugs-induced kidney injury is also one of the biggest causes of AKI (4). Future research in this area will help deepen our understanding of the immune regulation mechanism of MSCs therapy for AKI, and discover more potential therapeutic targets.

## Author Contributions

QH and XW had the original idea and wrote the first draft. HZ, JH, and GC reviewed the manuscript. HZ and XD provided critical input. All authors contributed to the article and approved the submitted version.

## Funding

This research was funded by The National Natural Science Foundation of China (No. 61971441) and the National Key R&D Program of China (No. 2016YFC1305500, 2018YFA0108803).

## Conflict of Interest

The authors declare that the research was conducted in the absence of any commercial or financial relationships that could be construed as a potential conflict of interest.

## References

[1] Hoste EAJ Kellum JA Selby NM Zarbock A Palevsky PM Bagshaw SM. Global Epidemiology and Outcomes of Acute Kidney Injury. Nat Rev Nephrol (2018) 14(10):607–25. 10.1038/s41581-018-0052-0 30135570

[2] Izzedine H Perazella MA. Anticancer Drug-Induced Acute Kidney Injury. Kidney Int Rep (2017) 2(4):504–14. 10.1016/j.ekir.2017.02.008 PMC5720534 29318217

[3] Rolland AL Garnier AS Meunier K Drablier G Briet M. Drug-Induced Acute Kidney Injury: A Study From the French Medical Administrative and the French National Pharmacovigilance Databases Using Capture-Recapture Method. J Clin Med (2021) 10(2):168. 10.3390/jcm10020168 PMC7824808 33418844

[4] Perazella MA. Drug-Induced Acute Kidney Injury: Diverse Mechanisms of Tubular Injury. Curr Opin Crit Care (2019) 25(6):550–7. 10.1097/MCC.0000000000000653 31483318

[5] Liu Y Fang J. Mesenchymal Stem Cells as Therapeutic Agents and Novel Carriers for the Delivery of Candidate Genes in Acute Kidney Injury. Stem Cells Int (2020) 2020:8875554. 10.1155/2020/8875554 33381189 PMC7748887

[6] Brown JR Rezaee ME Marshall EJ Matheny ME. Hospital Mortality in the United States Following Acute Kidney Injury. BioMed Res Int (2016) 2016:4278579. 10.1155/2016/4278579 27376083 PMC4916271

[7] Oh GS Kim HJ Shen A Lee SB Khadka D Pandit A. Cisplatin-Induced Kidney Dysfunction and Perspectives on Improving Treatment Strategies. Electrolyte Blood Press (2014) 12(2):55–65. 10.5049/ebp.2014.12.2.55 25606044 PMC4297704

[8] Volarevic V Djokovic B Jankovic MG Harrell CR Fellabaum C Djonov V. Molecular Mechanisms of Cisplatin-Induced Nephrotoxicity: A Balance on the Knife Edge Between Renoprotection and Tumor Toxicity. J BioMed Sci (2019) 26(1):25. 10.1186/s12929-019-0518-9 30866950 PMC6417243

[9] Zhou J An C Jin X Hu Z Safirstein RL Wang Y. TAK1 Deficiency Attenuates Cisplatin-Induced Acute Kidney Injury. Am J Physiol Renal Physiol (2020) 318(1):F209–15. 10.1152/ajprenal.00516.2019 PMC6985823 31813254

[10] Ozkok A Edelstein CL. Pathophysiology of Cisplatin-Induced Acute Kidney Injury. BioMed Res Int (2014) 2014:967826. 10.1155/2014/967826 25165721 PMC4140112

[11] Xu Y Ma H Shao J Wu J Zhou L Zhang Z. A Role for Tubular Necroptosis in Cisplatin-Induced Aki. J Am Soc Nephrol (2015) 26(11):2647–58. 10.1681/asn.2014080741 PMC4625668 25788533

[12] Mulay SR Linkermann A Anders HJ. Necroinflammation in Kidney Disease. J Am Soc Nephrol (2016) 27(1):27–39. 10.1681/asn.2015040405 26334031 PMC4696588

[13] Wu L Rong C Zhou Q Zhao X Zhuansun XM Wan S. Bone Marrow Mesenchymal Stem Cells Ameliorate Cisplatin-Induced Renal Fibrosis Via Mir-146a-5p/Tfdp2 Axis in Renal Tubular Epithelial Cells. Front Immunol (2020) 11:623693. 10.3389/fimmu.2020.623693 33664736 PMC7921314

[14] Qi J Xue Q Kuang L Xie L Luo R Nie X. Berberine Alleviates Cisplatin-Induced Acute Kidney Injury by Regulating Mitophagy Via PINK 1/Parkin Pathway. Transl Androl Urol (2020) 9(4):1712–24. 10.21037/tau-20-1129 PMC7475663 32944532

[15] Yang SK Han YC He JR Yang M Zhang W Zhan M. Mitochondria Targeted Peptide SS-31 Prevent on Cisplatin-Induced Acute Kidney Injury Via Regulating Mitochondrial ROS-NLRP3 Pathway. BioMed Pharmacother (2020) 130:110521. 10.1016/j.biopha.2020.110521 32717631

[16] Huang J Bao D Lei CT Tang H Zhang CY Su H. Selenoprotein T Protects Against Cisplatin-Induced Acute Kidney Injury Through Suppression of Oxidative Stress and Apoptosis. FASEB J (2020) 34(9):11983–96. 10.1096/fj.202000180RR 32686857

[17] Han SJ Lee HT. Mechanisms and Therapeutic Targets of Ischemic Acute Kidney Injury. Kidney Res Clin Pract (2019) 38(4):427–40. 10.23876/j.krcp.19.062 PMC6913588 31537053

[18] Ghane Shahrbaf F Assadi F. Drug-Induced Renal Disorders. J Renal Inj Prev (2015) 4(3):57–60. 10.12861/jrip.2015.12 26468475 PMC4594214

[19] Robert L Ficheur G Gautier S Servais A Luyckx M Soula J. Community-Acquired Acute Kidney Injury Induced By Drugs In Older Patients: A Multifactorial Event. Clin Interv Aging (2019) 14:2105–13. 10.2147/CIA.S217567 PMC6901120 31824141

[20] Dellepiane S Leventhal JS Cravedi P. T Cells and Acute Kidney Injury: A Two-Way Relationship. Front Immunol (2020) 11:1546. 10.3389/fimmu.2020.01546 32765535 PMC7379378

[21] Lemley KV Kriz W. Anatomy of the Renal Interstitium. Kidney Int (1991) 39(3):370–81. 10.1038/ki.1991.49 2062030

[22] Aggarwal BB. Signalling Pathways of the TNF Superfamily: A Double-Edged Sword. Nat Rev Immunol (2003) 3(9):745–56. 10.1038/nri1184 12949498

[23] Wang WW Wang Y Li K Tadagavadi R Friedrichs WE Budatha M. Il-10 From Dendritic Cells But Not From T Regulatory Cells Protects Against Cisplatin-Induced Nephrotoxicity. PloS One (2020) 15(9):e0238816. 10.1371/journal.pone.0238816 32898157 PMC7478814

[24] Zhang J Li Q Zou YR Wu SK Lu XH Li GS. Hmgb1-TLR4-IL-23-IL-17A Axis Accelerates Renal Ischemia-Reperfusion Injury Via the Recruitment and Migration of Neutrophils. Int Immunopharmacol (2021) 94:107433. 10.1016/j.intimp.2021.107433 33592404

[25] Faubel S Lewis EC Reznikov L Ljubanovic D Hoke TS Somerset H. Cisplatin-Induced Acute Renal Failure Is Associated With an Increase in the Cytokines Interleukin (IL)-1beta, IL-18, Il-6, and Neutrophil Infiltration in the Kidney. J Pharmacol Exp Ther (2007) 322(1):8–15. 10.1124/jpet.107.119792 17400889

[26] Fan Y Hao Y Gao D Gao L Li G Zhang Z. Phenotype and Function of Macrophage Polarization in Monocrotaline-Induced Pulmonary Arterial Hypertension Rat Model. Physiol Res (2021) 70(2):213–226. 10.33549/physiolres.934456 33676385 PMC8820576

[27] Tang M Zhang K Li Y He QH Li GQ Zheng QY. Mesenchymal Stem Cells Alleviate Acute Kidney Injury by Down-Regulating C5a/C5aR Pathway Activation. Int Urol Nephrol (2018) 50(8):1545–53. 10.1007/s11255-018-1844-7 29594894

[28] Ramesh G Reeves WB. TNF-Alpha Mediates Chemokine and Cytokine Expression and Renal Injury in Cisplatin Nephrotoxicity. J Clin Invest (2002) 110(6):835–42. 10.1172/jci15606 PMC151130 12235115

[29] Liu P Li X Lv W Xu Z. Inhibition of CXCL1-CXCR2 Axis Ameliorates Cisplatin-Induced Acute Kidney Injury by Mediating Inflammatory Response. BioMed Pharmacother (2020) 122:109693. 10.1016/j.biopha.2019.109693 31812015

[30] Andrade-Oliveira V Foresto-Neto O Watanabe IKM Zatz R Camara NOS. Inflammation in Renal Diseases: New and Old Players. Front Pharmacol (2019) 10:1192. 10.3389/fphar.2019.01192 31649546 PMC6792167

[31] Hu J Zhang L Cui S Zhu F Li D Feng Z. [Mesenchymal Stem Cells Attenuate Acute Kidney Injury Via Regulation of Natural Immune System]. Zhonghua Wei Zhong Bing Ji Jiu Yi Xue (2016) 28(3):235–40. 29917337

[32] Ceruso A Gonzalez-Pujana A Igartua M Santos-Vizcaino E Hernandez RM. Latest Advances to Enhance the Therapeutic Potential of Mesenchymal Stromal Cells for the Treatment of Immune-Mediated Diseases. Drug Delivery Transl Res (2021) 11(2):498–514. 10.1007/s13346-021-00934-5 33634433

[33] Wright A Arthaud-Day ML Weiss ML. Therapeutic Use of Mesenchymal Stromal Cells: The Need for Inclusive Characterization Guidelines to Accommodate All Tissue Sources and Species. Front Cell Dev Biol (2021) 9:632717. 10.3389/fcell.2021.632717 33665190 PMC7921162

[34] Yoo KH Jang IK Lee MW Kim HE Yang MS Eom Y. Comparison of Immunomodulatory Properties of Mesenchymal Stem Cells Derived From Adult Human Tissues. Cell Immunol (2009) 259(2):150–6. 10.1016/j.cellimm.2009.06.010 19608159

[35] El-Sayed M El-Feky MA El-Amir MI Hasan AS Tag-Adeen M Urata Y. Immunomodulatory Effect of Mesenchymal Stem Cells: Cell Origin and Cell Quality Variations. Mol Biol Rep (2019) 46(1):1157–65. 10.1007/s11033-018-04582-w 30628022

[36] Ribeiro A Laranjeira P Mendes S Velada I Leite C Andrade P. Mesenchymal Stem Cells From Umbilical Cord Matrix, Adipose Tissue and Bone Marrow Exhibit Different Capability to Suppress Peripheral Blood B, Natural Killer and T Cells. Stem Cell Res Ther (2013) 4(5):125. 10.1186/scrt336 24406104 PMC3854702

[37] Wegmeyer H Broske AM Leddin M Kuentzer K Nisslbeck AK Hupfeld J. Mesenchymal Stromal Cell Characteristics Vary Depending on Their Origin. Stem Cells Dev (2013) 22(19):2606–18. 10.1089/scd.2013.0016 PMC3780294 23676112

[38] Heo JS Choi Y Kim HS Kim HO. Comparison of Molecular Profiles of Human Mesenchymal Stem Cells Derived From Bone Marrow, Umbilical Cord Blood, Placenta and Adipose Tissue. Int J Mol Med (2016) 37(1):115–25. 10.3892/ijmm.2015.2413 PMC4687432 26719857

[39] Bochev I Elmadjian G Kyurkchiev D Tzvetanov L Altankova I Tivchev P. Mesenchymal Stem Cells From Human Bone Marrow or Adipose Tissue Differently Modulate Mitogen-Stimulated B-cell Immunoglobulin Production In Vitro. Cell Biol Int (2008) 32(4):384–93. 10.1016/j.cellbi.2007.12.007 18262807

[40] Li G Zhang XA Wang H Wang X Meng CL Chan CY. Comparative Proteomic Analysis of Mesenchymal Stem Cells Derived From Human Bone Marrow, Umbilical Cord, and Placenta: Implication in the Migration. Proteomics (2009) 9(1):20–30. 10.1002/pmic.200701195 19116983

[41] Barcia RN Santos JM Filipe M Teixeira M Martins JP Almeida J. What Makes Umbilical Cord Tissue-Derived Mesenchymal Stromal Cells Superior Immunomodulators When Compared to Bone Marrow Derived Mesenchymal Stromal Cells? Stem Cells Int (2015) 2015:583984. 10.1155/2015/583984 26064137 PMC4443932

[42] Li CY Wu XY Tong JB Yang XX Zhao JL Zheng QF. Comparative Analysis of Human Mesenchymal Stem Cells From Bone Marrow and Adipose Tissue Under Xeno-Free Conditions for Cell Therapy. Stem Cell Res Ther (2015) 6:55. 10.1186/s13287-015-0066-5 25884704 PMC4453294

[43] Baksh D Yao R Tuan RS. Comparison of Proliferative and Multilineage Differentiation Potential of Human Mesenchymal Stem Cells Derived From Umbilical Cord and Bone Marrow. Stem Cells (2007) 25(6):1384–92. 10.1634/stemcells.2006-0709 17332507

[44] Cantaluppi V Biancone L Quercia A Deregibus MC Segoloni G Camussi G. Rationale of Mesenchymal Stem Cell Therapy in Kidney Injury. Am J Kidney Dis (2013) 61(2):300–9. 10.1053/j.ajkd.2012.05.027 22938846

[45] Fontaine MJ Shih H Schäfer R Pittenger MF. Unraveling the Mesenchymal Stromal Cells’ Paracrine Immunomodulatory Effects. Transfus Med Rev (2016) 30(1):37–43. 10.1016/j.tmrv.2015.11.004 26689863

[46] Muller L Tunger A Wobus M von Bonin M Towers R Bornhauser M. Immunomodulatory Properties of Mesenchymal Stromal Cells: An Update. Front Cell Dev Biol (2021) 9:637725. 10.3389/fcell.2021.637725 33634139 PMC7900158

[47] Peired AJ Sisti A Romagnani P. Mesenchymal Stem Cell-Based Therapy for Kidney Disease: A Review of Clinical Evidence. Stem Cells Int (2016) 2016:4798639. 10.1155/2016/4798639 27721835 PMC5046016

[48] Najar M Raicevic G Fayyad-Kazan H Bron D Toungouz M Lagneaux L. Mesenchymal Stromal Cells and Immunomodulation: A Gathering of Regulatory Immune Cells. Cytotherapy (2016) 18(2):160–71. 10.1016/j.jcyt.2015.10.011 26794710

[49] Jang HR Rabb H. Immune Cells in Experimental Acute Kidney Injury. Nat Rev Nephrol (2015) 11(2):88–101. 10.1038/nrneph.2014.180 25331787

[50] Huen SC Cantley LG. Macrophage-Mediated Injury and Repair After Ischemic Kidney Injury. Pediatr Nephrol (2015) 30(2):199–209. 10.1007/s00467-013-2726-y 24442822 PMC5048744

[51] Holditch SJ Brown CN Lombardi AM Nguyen KN Edelstein CL. Recent Advances in Models, Mechanisms, Biomarkers, and Interventions in Cisplatin-Induced Acute Kidney Injury. Int J Mol Sci (2019) 20(12):3011. 10.3390/ijms20123011 PMC6627318 31226747

[52] Tadagavadi RK Reeves WB. Renal Dendritic Cells Ameliorate Nephrotoxic Acute Kidney Injury. J Am Soc Nephrol (2010) 21(1):53–63. 10.1681/asn.2009040407 19875815 PMC2799272

[53] Zhang B Ramesh G Uematsu S Akira S Reeves WB. TLR4 Signaling Mediates Inflammation and Tissue Injury in Nephrotoxicity. J Am Soc Nephrol (2008) 19(5):923–32. 10.1681/asn.2007090982 PMC2386719 18256356

[54] Liu M Chien CC Burne-Taney M Molls RR Racusen LC Colvin RB. A Pathophysiologic Role for T Lymphocytes in Murine Acute Cisplatin Nephrotoxicity. J Am Soc Nephrol (2006) 17(3):765–74. 10.1681/asn.2005010102 16481417

[55] Zhang J Rudemiller NP Patel MB Wei Q Karlovich NS Jeffs AD. Competing Actions of Type 1 Angiotensin Ii Receptors Expressed on T Lymphocytes and Kidney Epithelium During Cisplatin-Induced Aki. J Am Soc Nephrol (2016) 27(8):2257–64. 10.1681/asn.2015060683 PMC4978052 26744488

[56] Abumaree MH Abomaray FM Alshabibi MA AlAskar AS Kalionis B. Immunomodulatory Properties of Human Placental Mesenchymal Stem/Stromal Cells. Placenta (2017) 59:87–95. 10.1016/j.placenta.2017.04.003 28411943

[57] Snelgrove SL Lo C Hall P Lo CY Alikhan MA Coates PT. Activated Renal Dendritic Cells Cross Present Intrarenal Antigens After Ischemia-Reperfusion Injury. Transplantation (2017) 101(5):1013–24. 10.1097/tp.0000000000001427 27495751

[58] Dong X Swaminathan S Bachman LA Croatt AJ Nath KA Griffin MD. Resident Dendritic Cells Are the Predominant TNF-secreting Cell in Early Renal Ischemia-Reperfusion Injury. Kidney Int (2007) 71(7):619–28. 10.1038/sj.ki.5002132 17311071

[59] Tadagavadi RK Reeves WB. Endogenous IL-10 Attenuates Cisplatin Nephrotoxicity: Role of Dendritic Cells. J Immunol (2010) 185(8):4904–11. 10.4049/jimmunol.1000383 PMC3169908 20844196

[60] Simovic Markovic B Gazdic M Arsenijevic A Jovicic N Jeremic J Djonov V. Mesenchymal Stem Cells Attenuate Cisplatin-Induced Nephrotoxicity in iNOS-Dependent Manner. Stem Cells Int (2017) 2017:1315378. 10.1155/2017/1315378 28828008 PMC5554561

[61] Scutera S Salvi V Lorenzi L Piersigilli G Lonardi S Alotto D. Adaptive Regulation of Osteopontin Production by Dendritic Cells Through the Bidirectional Interaction With Mesenchymal Stromal Cells. Front Immunol (2018) 9:1207. 10.3389/fimmu.2018.01207 29910810 PMC5992779

[62] Jiang XX Zhang Y Liu B Zhang SX Wu Y Yu XD. Human Mesenchymal Stem Cells Inhibit Differentiation and Function of Monocyte-Derived Dendritic Cells. Blood (2005) 105(10):4120–6. 10.1182/blood-2004-02-0586 15692068

[63] Nauta AJ Kruisselbrink AB Lurvink E Willemze R Fibbe WE. Mesenchymal Stem Cells Inhibit Generation and Function of Both CD34+-derived and Monocyte-Derived Dendritic Cells. J Immunol (2006) 177(4):2080–7. 10.4049/jimmunol.177.4.2080 16887966

[64] English K Barry FP Mahon BP. Murine Mesenchymal Stem Cells Suppress Dendritic Cell Migration, Maturation and Antigen Presentation. Immunol Lett (2008) 115(1):50–8. 10.1016/j.imlet.2007.10.002 18022251

[65] Djouad F Charbonnier LM Bouffi C Louis-Plence P Bony C Apparailly F. Mesenchymal Stem Cells Inhibit the Differentiation of Dendritic Cells Through an interleukin-6-dependent Mechanism. Stem Cells (2007) 25(8):2025–32. 10.1634/stemcells.2006-0548 17510220

[66] Chiesa S Morbelli S Morando S Massollo M Marini C Bertoni A. Mesenchymal Stem Cells Impair In Vivo T-cell Priming by Dendritic Cells. Proc Natl Acad Sci USA (2011) 108(42):17384–9. 10.1073/pnas.1103650108 PMC3198360 21960443

[67] Gazdic M Volarevic V Arsenijevic N Stojkovic M. Mesenchymal Stem Cells: A Friend or Foe in Immune-Mediated Diseases. Stem Cell Rev Rep (2015) 11(2):280–7. 10.1007/s12015-014-9583-3 25592610

[68] Bolisetty S Agarwal A. Neutrophils in Acute Kidney Injury: Not Neutral Any More. Kidney Int (2009) 75(7):674–6. 10.1038/ki.2008.689 19282858

[69] Miao N Yin F Xie H Wang Y Xu Y Shen Y. The Cleavage of Gasdermin D by caspase-11 Promotes Tubular Epithelial Cell Pyroptosis and Urinary IL-18 Excretion in Acute Kidney Injury. Kidney Int (2019) 96(5):1105–20. 10.1016/j.kint.2019.04.035 31405732

[70] Luo CJ Zhang FJ Zhang L Geng YQ Li QG Hong Q. Mesenchymal Stem Cells Ameliorate Sepsis-Associated Acute Kidney Injury in Mice. Shock (2014) 41(2):123–9. 10.1097/shk.0000000000000080 24169208

[71] Matsumoto M Tanaka T Kaisho T Sanjo H Copeland NG Gilbert DJ. A Novel LPS-inducible C-Type Lectin is a Transcriptional Target of NF-IL6 in Macrophages. J Immunol (1999) 163(9):5039–48. 10528209

[72] Ichioka M Suganami T Tsuda N Shirakawa I Hirata Y Satoh-Asahara N. Increased Expression of Macrophage-Inducible C-type Lectin in Adipose Tissue of Obese Mice and Humans. Diabetes (2011) 60(3):819–26. 10.2337/db10-0864 PMC3046842 21282371

[73] Lu LH Oh DJ Dursun B He Z Hoke TS Faubel S. Increased Macrophage Infiltration and Fractalkine Expression in Cisplatin-Induced Acute Renal Failure in Mice. J Pharmacol Exp Ther (2008) 324(1):111–7. 10.1124/jpet.107.130161 17932247

[74] Rogers NM Ferenbach DA Isenberg JS Thomson AW Hughes J. Dendritic Cells and Macrophages in the Kidney: A Spectrum of Good and Evil. Nat Rev Nephrol (2014) 10(11):625–43. 10.1038/nrneph.2014.170 PMC4922410 25266210

[75] Cao Q Wang Y Harris DC. Pathogenic and Protective Role of Macrophages in Kidney Disease. Am J Physiol Renal Physiol (2013) 305(1):F3–11. 10.1152/ajprenal.00122.2013 23637206 PMC3725671

[76] Recknagel S Bindl R Kurz J Wehner T Schoengraf P Ehrnthaller C. C5aR-antagonist Significantly Reduces the Deleterious Effect of a Blunt Chest Trauma on Fracture Healing. J Orthop Res (2012) 30(4):581–6. 10.1002/jor.21561 PMC3244519 21922535

[77] Huang S You J Wang K Li Y Zhang Y Wei H. N-Acetylcysteine Attenuates Cisplatin-Induced Acute Kidney Injury by Inhibiting the C5a Receptor. BioMed Res Int (2019) 2019:4805853. 10.1155/2019/4805853 31111056 PMC6487137

[78] Ullah M Liu D Rai S Concepcion W Thakor A. Hsp70-Mediated NLRP3 Inflammasome Suppression Underlies Reversal of Acute Kidney Injury Following Extracellular Vesicle and Focused Ultrasound Combination Therapy. Int J Mol Sci (2020) 21(11):4085. 10.3390/ijms21114085 PMC7312940 32521623

[79] Ullah M Liu D Rai S Dadhania A Jonnakuti S Concepcion W. Reversing Acute Kidney Injury Using Pulsed Focused Ultrasound and MSC Therapy: A Role for HSP-Mediated Pi3k/Akt Signaling. Mol Ther Methods Clin Dev (2020) 17:683–94. 10.1016/j.omtm.2020.03.023 PMC7177168 32346546

